# Does repeated daytime napping over consecutive days enhance physical, perceptual, and cardiac autonomic responses in high-level adolescent basketball players?

**DOI:** 10.5114/biolsport.2026.159567

**Published:** 2026-04-13

**Authors:** Mehdi J Souabni, Maher Souabni, Omar Hammouda, Mathieu Nedelec, Achraf Ammar, Tarak Driss

**Affiliations:** 1 Interdisciplinary Laboratory in Neurosciences, Physiology and Psychology: Physical Activity, Health and Learning (LINP2), UFR STAPS (Faculty of Sport Sciences), Paris Nanterre University, Nanterre, France; 2 LBEPS, Univ Evry, IRBA, Université Paris-Saclay, Evry, France; 3 Research Laboratory, Molecular Bases of Human Pathology, LR19ES13, Faculty of Medicine, University of Sfax, Sfax, Tunisia; 4 Laboratory of Sport, Expertise and Performance (EA 7370), French National Institute of Sport (INSEP), Paris, France; 5 High Institute of Sport and Physical Education of Sfax, University of Sfax, Sfax 3000, Tunisia; 6 Department of Training and Movement Science, Institute of Sport Science, Johannes Gutenberg-University Mainz, 55122 Mainz, Germany; 7 Department of Nutrition and Food Technology, School of Agriculture, The University of Jordan, Amman, Jordan

**Keywords:** Adolescent athletes, Basketball, Congested training schedule, Repeated napping, sleep, Physical performance, Heart rate variability

## Abstract

Adolescent athletes experience insufficient sleep, impacting their health and performance. Napping has emerged as a promising sleep management strategy to counteract these consequences, with acute benefits on performance. However, the impact of repeated napping remains unclear. This study examined the effects of a five-day micro-cycle of 60-min daytime nap opportunities on physical performance, as well as perceptual and physiological responses in high-level adolescent athletes. Twelve high-level male adolescent basketball players (15.75 ± 0.62 years) participated in a randomized, counterbalanced crossover study with two conditions: five consecutive napping days (NAP) and five consecutive control (no napping) days (CON). Day- and night-time sleep was monitored via actigraphy, while perceptual (Hooper questionnaire) and physiological (Heart Rate Variability, HRV) responses were assessed at rest before and after the nap and control conditions across five consecutive days. Basketball-specific performances including offensive and defensive agility, upper body power, repeated jumps (RJ), and rating of perceived exertion (RPE) after RJ were evaluated 90-min following both conditions on days 1 and 5. NAP increased 24-h total sleep time (p = 0.003, d = 0.40), with longer and betterquality naps toward the end of the micro-cycle. Napping was associated with an increase in total HRV power, and a decrease in subjective fatigue, muscle soreness and RPE (0.002 ≤ p ≤ 0.035, 0.28 ≤ d ≤ 2.10). Five consecutive days of napping improved agility and jump performance (0.001 < p ≤ 0.015, 0.83 ≤ d ≤ 1.61). Repeated napping helped to meet sleep recommendations, improved perceptual and physiological responses, and sport-specific abilities, highlighting its relevance as a practical strategy to support recovery and well-being in adolescent athletes, particularly during training camps.

## INTRODUCTION

Sleep serves as the gold standard recovery strategy during training periods and competitions [1]. During adolescence, sleep quality and quantity undergo marked changes due to the interaction of (i) maturational development, characterized by a circadian phase delay and reduced sleep pressure, and (ii) societal and psychosocial factors such as increased screen exposure and early school start times. Together, these factors contribute to shorter sleep duration, poorer sleep quality, and altered sleep patterns [2, 3]. Adolescent athletes are known to face combined academic and sport-related demands that often restrict sleep duration and impair recovery [4]. Importantly, they experience inadequate sleep even on weekends and holidays, when the general adolescent population typically compensates for weekday sleep debt [5]. In this way, several studies focusing on athletes’ sleep reported that younger athletes tend to have poorer sleep quality, sleep less, and experience lower sleep efficiency (SE) compared to adults athletes [6, 7]. A recent review reported that adolescent athletes typically obtain less than the recommended 8–10 h of sleep per night and exhibit longer sleep onset latency than adolescent non-athletes [8].

Moreover, high training loads and congested training periods can markedly disrupt athletes’ sleep, with intensive micro-cycles and training camps associated with longer sleep onset latency, reduced sleep efficiency, increased nocturnal awakenings, and greater sleep fragmentation index (SFI) [9, 10]. Additionally, physical discomfort related to intense training, including muscle soreness and pain, may further impair sleep and recovery [11]. Combined with maturational and psychosocial factors, these sport-related constraints can substantially reduce sleep quantity and quality, increasing injury risk in adolescent athletes [8] and negatively affecting performance, notably in basketball [12]. For these reasons, identifying strategies to supplement young athletes’ sleep while optimizing performance is of paramount importance.

Interestingly, sleep management approaches, especially napping, have been emphasized by sports scientists [13–16] due to the positive impact of this approach on physical [17] and cognitive [15] performance, as well as physiological responses [18, 19]. Especially during micro-cycles of intensive training, it has been suggested to emphasize sufficient sleep periods or even provide extra sleep opportunities in order to optimize athletes’ recovery [10]. Thornton et al. [20] reported that daytime naps (≈30 min) may be beneficial in compensating for jeopardized nocturnal sleep during a two-week high-intensity training camp. Specifically in adolescent athletes, daytime napping has been shown to effectively complement reduced nighttime sleep, as demonstrated by a study showing similar TST across three conditions (i.e., 9 h of nocturnal sleep, 8 h of nocturnal sleep +1 h nap, and 7 h of nocturnal sleep +2 h nap) [21].

While several studies have examined the acute effects of napping in adolescent athletes [22–24], findings on its impact on physical performance remain inconclusive. In contrast, the effects of repeated daytime napping across a micro-cycle have received comparatively little attention in this population. Evidence from non-athlete adolescents suggests that a five-day period of repeated daytime naps, particularly with 60-min nap opportunities, can enhance cognitive performance [25]. Such a duration is considered sufficient to allow the occurrence of slow-wave sleep and to support recovery-related processes [13], while remaining compatible with adolescent athletes’ routines and unlikely to compromise nocturnal sleep, especially among adolescents experiencing habitual sleep restriction [25]. Accordingly, the present study aimed to examine the effects of a fiveday micro-cycle of 60-min daytime nap opportunities on physical performance, perceptual and physiological responses in adolescent athletes. We hypothesize that daytime napping would be associated with improvements in physical outcomes as well as perceptual and physiological parameters on Day 1, with these effects becoming more pronounced following the five-day micro-cycle.

## MATERIALS AND METHODS

### Participants

Twelve high-level male adolescent basketball players (age: 15.75 ± 0.62 years, height: 1.82 ± 0.07 m, weight: 71.36 ± 12.75 kg, fat percentage: 16.49 ± 8.54 % and BMI: 21.53 ± 2.62 kg/m^2^) including six international-level athletes, were recruited. All were high school students training 5 times/week (2 h/session) and playing one weekly match. Inclusion criteria excluded professionally diagnosed sleep disorders, illness, inflammation, pain, medication affecting sleep and/or HRV, habitual napping (≥ 1 nap/week), extreme chronotypes, and nicotine and alcohol consumption. Only caffeine naïve athletes (< 80 mg · day-1) [26] were recruited and caffeine consumption was not allowed during the study. Participants’ habitual sleep was monitored for one week, averaging a TST of 6.56 ± 0.81 h and an SE of 81.30 ± 5.31%, and Pittsburgh sleep quality index (PSQI) [27], Morningness–eveningness questionnaire (MEQ) [28], and Cleveland adolescent sleepiness questionnaire (CASQ) [29] were used to assess the subjective sleep quality (4.67 ± 1.30), chronotype (48.25 ± 9.14) and daytime sleepiness (37.08 ± 9.41), respectively. Sample size was determined a priori using G*Power [30], with an effect size of 0.54 [17], yielding a minimum of nine participants for 95% power. Ethical approval was granted (CPP SUD No 0339/2021), and written informed consent was obtained from all participants’ parents/guardians, in accordance with the Declaration of Helsinki.

### Procedure

**FIG. 1:**
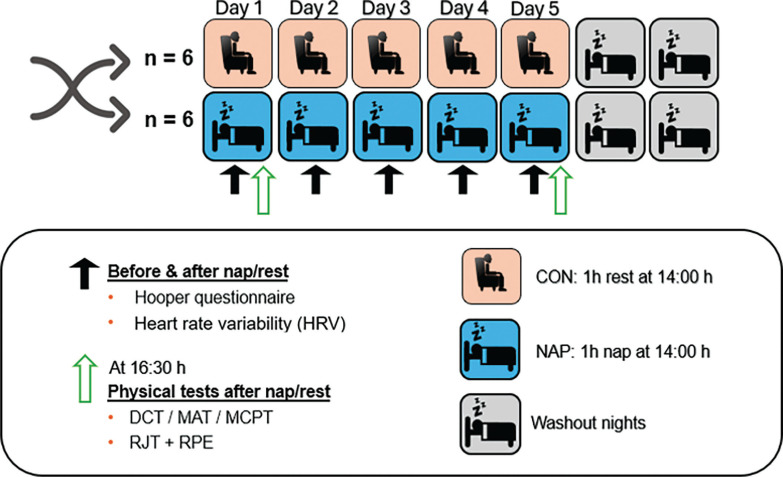
Experimental protocol. DCT: Dribble control test. MAT: Modified agility T-test. MCPT: Medicine ball chest pass test. RJT: sixrepeated jump test and RPE: Rating of perceived exertion.

The experimental design is presented in Figure 1. To minimize learning and adaptation biases, participants underwent a familiarization session involving exposure to the laboratory setting, instrumentation, nap environment, and study procedures.

The study, conducted during a training camp, followed a randomized, counterbalanced crossover design with two conditions: five consecutive days of 60-min daytime nap opportunities (NAP) and five consecutive days of control condition (CON), following normal sleep nights and separated by a 2-day washout without napping. Each day, participants had a standardized breakfast (09:00 h), a shooting session (10:00–11:30 h), and an isocaloric lunch (12:30 h). At 14:00 h, participants either napped for one hour in a dark, quiet room (NAP) or remained seated in a comfortable chair doing passive activities (i.e., reading, watching neutral television programs, playing card games) for the same duration (CON). The Hooper questionnaire and heart rate variability (HRV) were assessed each day pre- and post-nap/rest. Sleep was monitored via actigraphy. Physical and sport-specific tests were conducted on Days 1 and 5 of each week at 16:30 h, 90 min after awakening from the nap to minimize potential sleep inertia effects. Following a standardized warm-up (5-min self-paced jogging, 10-min dynamic stretching, and five progressive sprints), participants completed a test battery comprising the dribble control test (DCT), modified agility T-test (MAT), medicine ball chest pass test (MCPT), and six-repeated jump test (RJT), with RPE recorded after the RJT. Tests were performed in the same order.

### Measured variables

#### Sleep measurement

Objective measurements of sleep quantity and quality during nocturnal sleep and naps were obtained using actigraphy, a validated method for monitoring rest-activity cycles in adolescents [31]. Participants wore a GT3X actigraph (Actigraph, Pensacola, FL, USA) on their non-dominant wrist throughout the study. Key sleep parameters (i.e., Time in bed (TIB), Total sleep time (TST), Sleep efficiency (SE), Sleep onset latency (SOL), Wake after sleep onset (WASO), Sleep fragmentation index (SFI)) were extracted and analyzed using Actilife software (version 6.13.7, Actigraph, Pensacola, FL, USA), with data cross-checked and adjusted using participant sleep diaries.

#### Subjective measurements

In addition to PSQI, MEQ and CASQ questionnaires administrated in the familiarization session, the Hooper questionnaire, a self-assessment tool, evaluated athletes’ recovery and readiness for training through four items (sleep quality, stress, fatigue, muscle soreness), each rated on a 7-point Likert scale [32]. RPE scores were collected after RJT using Borg’s 15-point scale (6–20) [33].

#### Physiological parameters

Heart rate (HR) was monitored via a chest sensor (Mooky Center technology, HR5) [34], synchronized with an ActiGraph GT3X to record HR and R-R intervals. Following Task Force guidelines for recording short-term HRV [35], participants were instructed to lie still in a supine position, avoid verbal communication, and maintain a natural breathing rate during recordings, which were performed before and immediately after each nap/control condition. R-R data were extracted with ActiLife (v6.13.7) and analyzed using Kubios HRV Scientific (v3.5, Matlab, Finland), with correction of ectopic beats. HR mean, minimum and peak were expressed relative to HRmax using Tanaka formula [36]. HRV analysis included timedomain parameters: the standard deviation of the normal R-R interval (SDNN), the root mean square of the successive differences in the R-R intervals (RMSSD), and the percentage of successive interval differences larger than 50 ms (PNN50). Frequency-domain parameters were derived through fast Fourier transformation, quantifying the low-frequency bands (LF) (0.04–0.15 Hz) and high frequency (HF) (0.15–0.40 Hz) expressed in normalized units (nu), and the LF/HF ratio, as well as total power (TP), a representation of total HRV in the frequency domain. Additionally, the HRV triangular index (HRV index), a geometric measure reflecting global HRV, and the stress index (SI) were calculated. A 5-min epoch is conventionally used to study the above-mentioned variables [37].

#### Basketball-specific physical performance

Offensive agility (OA) was assessed using the DCT, a valid measure of basketball-specific agility [38]. The task involves dribbling a ball as quickly as possible while changing hands and direction around five cones placed within a restricted area. Defensive agility (DA) was evaluated using the MAT, a reliable measure incorporating basketballspecific footwork [39] successfully used with male adolescent basketball players [40]. The protocol involved sprinting 5 m forward, shuffling 2.5 m left, 5 m right, 2.5 m left again, and backpedaling to the starting line. For agility tests, performance was timed (in seconds) using photocell technology (Wireless Training Timer, Microgate Witty, Tesma Sport, Kranj, Slovenia) to ensure precision and reliability. Upper body power (UBP) was measured using MCPT. Participants sat against a wall with legs extended horizontally to ensure consistency in technique and executed a 2-handed Chest Pass, and throw distance was recorded in meters [17]. Repeated jumping performance (RJ) was evaluated using a six-repeated jump test (RJT), based on the validated protocol by Meckel et al. [41] for adolescent basketball players. Participants performed six consecutive maximal vertical jumps using the free countermovement jump technique on an Optojump Next system (v1.12.23.0, Microgate, Italy), with 30 seconds of rest between trials, and jump height and power output were recorded. For all tests, the mean value of three trials was used for analysis.

### Statistical analyses

Data were analyzed using SPSS Statistics (v.25, IBM, NY, USA). Normality was assessed using the Shapiro–Wilk test. When normally distributed, a three-way repeated-measures ANOVA [5 days × 2 conditions (NAP, CON) × 2 times (pre, post)] was used for Hooper global score and HRV parameters, and a two-way repeated-measures ANOVA [2 days × 2 conditions] for OA, DA, UBP, RJ, and RPE, and a one-way repeated-measures ANOVA [5 days] for napping sleep parameters. Bonferroni post hoc tests followed significant ANOVA effects. Effect sizes were reported as partial eta squared (ηp^2^: small = 0.01, medium = 0.06, large = 0.14). Nonnormally distributed Hooper subscales (sleep, stress, fatigue, soreness) were analyzed via Friedman’s ANOVA with Wilcoxon post hoc tests. Paired t-tests or Wilcoxon tests (as appropriate) were used to compare nocturnal sleep parameters and TST over 24 h between conditions. Cohen’s *d* was used to report effect sizes, classified as small (*d* < 0.5), moderate (0.5 ≤ *d* < 0.8), and large (*d* ≥ 0.8) [42]. Statistical significance was set at p < 0.05.

## RESULTS

### Objective sleep measures

**FIG. 2:**
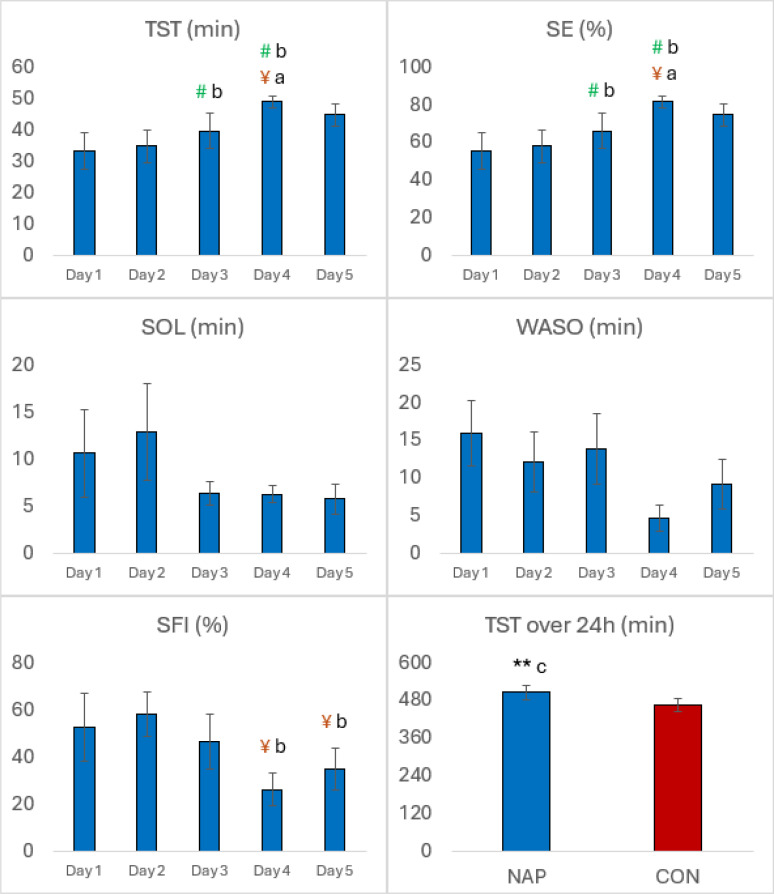
Mean ± standard error of sleep parameters during naps across five days and total sleep time (TST) over 24 hours averaged over five days in NAP and CON conditions. CON: Five consecutive days of rest (control condition). NAP: Five consecutive days of napping. SE: Sleep efficiency. SFI: Sleep fragmentation index. SOL: Sleep onset latency. TIB: Time in bed. TST: Total sleep time and WASO: Wake after sleep onset. Significant difference compared to Day 1, # p < 0.05. Significant difference compared to Day 2, ¥p < 0.05. Significant difference compared to CON, **p < 0.01. a, b and c indicate large, medium and small Cohen’s *d* effect size, respectively.

Actigraphy showed that, during the 5-day period, athletes averaged 40.35 ± 16.59 min of sleep during naps with 67.25 ± 27.65% of SE, 8.43 ± 11.19 min of SOL and 11.22 ± 13.17 min of WASO. Naps’ sleep parameters (i.e., TST, SE, SFI, SOL and WASO) across the five days are detailed in Figure 2. TST during the nap was significantly higher on Day 3 and 4 compared to Day 1 (p = 0.045 and p = 0.025, respectively), and on Day 4 compared to Day 2 (p = 0.012). In addition, SFI decreased significantly on Day 4 and 5 compared to Day 2 (p = 0.045 and p = 0.042, respectively). Furthermore, the 5-day average of TST over 24 h was significantly longer in the NAP condition than in CON (p = 0.003) (Figure 2). Night-time sleep parameters before (Day 0) and during the experimental period (Days 1–5) are presented in Table 1.

**TABLE 1:** Sleep parameters (mean ± SD) during the nights before (Day 0) and during experimental days (Day 1, 2, 3, 4 and 5) in NAP and CON conditions.

Sleep parameters	Days	NAP	CON	p-value	Cohen’s *d*
TIB (min)	Day 0	571.67 ± 54.88	531.83 ± 65.01	0.13	0.47
	Day 1	564.42 ± 59.49	557.42 ± 63.00	0.76	0.09
	Day 2	542.42 ± 72.89	543.25 ± 82.99	0.98	0.01
	Day 3	530.67 ± 89.22	497.17 ± 84.63	0.36	0.28
	Day 4	512.00 ± 70.24	557.33 ± 73.25	0.13	0.47
	Day 5	527.50 ± 90.72	536.67 ± 81.19	0.74	0.10

TST (min)	Day 0	502.17 ± 53.44	465.83 ± 69.73	0.17	0.42
	Day 1	490.17 ± 39.35	488.17 ± 56.66	0.92	0.03
	Day 2	478.33 ± 75.2	465.92 ± 69.35	0.56	0.17
	Day 3	456.58 ± 83.04	425.67 ± 73.86	0.38	0.26
	Day 4	447.83 ± 63.51	493.08 ± 74.15	0.10	0.52
	Day 5	451.67 ± 85.94	458.83 ± 67.77	0.79	0.10

SE (%)	Day 0	87.90 ± 5.10	87.4 ± 4.96	0.82	0.07
	Day 1	87.13 ± 4.22	87.7 ± 5.13	0.65	0.14
	Day 2	88.31 ± 7.93	85.98 ± 5.77	0.48	0.27
	Day 3	86.14 ± 6.48	85.83 ± 5.73	0.88	0.04
	Day 4	87.62 ± 5.67	88.32 ± 3.85	0.74	0.10
	Day 5	85.68 ± 6.68	85.73 ± 6.13	0.98	0.01

WASO (min)	Day 0	64.83 ± 31.72	56.42 ± 21.39	0.40	0.25
	Day 1	69.67 ± 28.08	56.17 ± 18.66	0.07	0.58
	Day 2	58.75 ± 42.18	62.58 ± 29.15	0.84	0.06
	Day 3	68.00 ± 33.67	63.08 ± 30.9	0.61	0.15
	Day 4	58.17 ± 32.04	57.08 ± 18.11	0.97	0.03
	Day 5	70.25 ± 33.99	64.33 ± 31.17	0.61	0.15

SOL (min)	Day 0	4.67 ± 3.58	9.58 ± 7.86	**0.03**	**0.72**
	Day 1	4.58 ± 5.90	13.08 ± 19.74	0.31	0.38
	Day 2	5.33 ± 4.89	14.75 ± 14.6	**0.02**	**0.87**
	Day 3	6.08 ± 6.33	8.42 ± 7.88	0.67	0.19
	Day 4	6.00 ± 7.54	7.17 ± 6.93	0.69	0.15
	Day 5	5.58 ± 6.14	13.5 ± 15.30	0.12	0.55

Abbreviations: CON: Five consecutive days of rest (control condition). NAP: Five consecutive days of napping. SE: Sleep efficiency. SOL: Sleep onset latency. TIB: Time in bed. TST: Total sleep time and WASO: Wake after sleep onset.

### Subjective measurements

#### Hooper

**FIG. 3:**
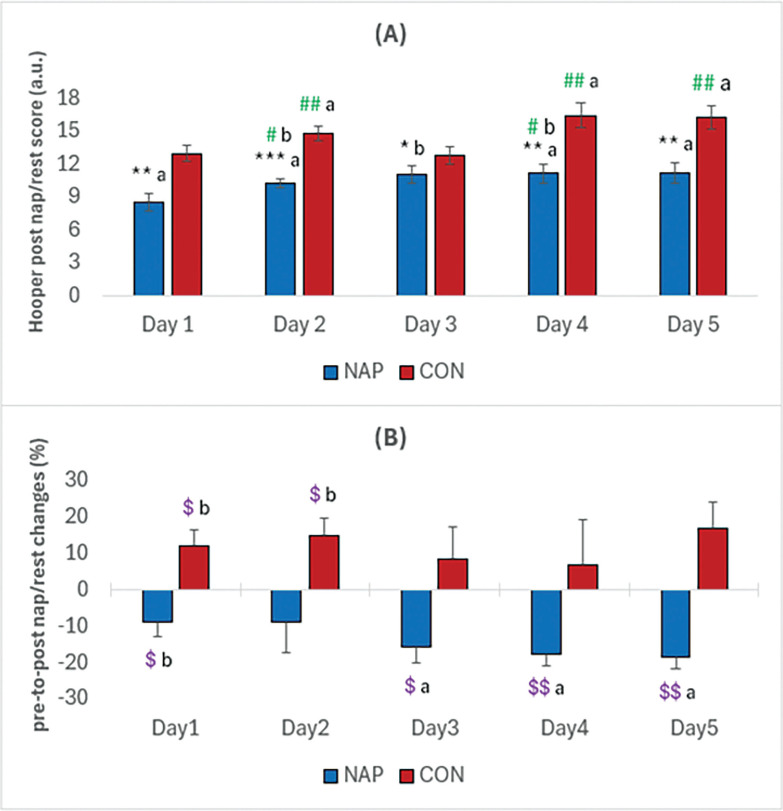
**(A)** Mean ± standard error of Hooper’s post nap/rest global scores across the five days. CON: Five consecutive days of rest (control condition). NAP: Five consecutive days of napping. Significant difference compared to CON, *p < 0.05, **p < 0.01, ***p < 0.001. Significant difference compared to Day 1, #p < 0.05. ##p < 0.01. (B) Percentage ± standard error of pre-to-post score changes for Hooper’s wellbeing index. Significant difference between pre- and post- nap/rest, $p < 0.05, $$p < 0.01. a and b indicate large and medium Cohen’s *d* effect size, respectively.

ANOVA revealed significant main effects of day (F_(4,44)_ = 9.99, p < 0.001, ηp^2^ = 0.48) and condition (F_(1,11)_ = 87.06, p < 0.001, ηp^2^ = 0.89), for the Hooper global score. Additionally, significant interactions (day × condition) (F_(4,44)_ = 3.27, p = 0.02, ηp^2^ = 0.23) and (condition × time) (F_(1,11)_ = 53.16, p < 0.001, ηp^2^ = 0.83) were found. Post hoc tests showed a significantly lower global score in post-nap compared to post-rest across all five days (0.0001 ≤ p ≤ 0.040) (Figure 3). Further, Hooper’s scores increased in CON from pre- to post-rest on Days 1 and 2 (p = 0.024 for both). In contrast, in NAP, post-nap scores were significantly lower than pre-nap values on Days 1, 3, 4, and 5 (0.001 ≤ p ≤ 0.034), but not on Day 2 (p = 0.197). Pre-to-post changes in Hooper’s global score for both conditions across the five-day period are shown in Figure 3. Furthermore, statistical analyses revealed a significant improvement in Hooper’s sleep quality, fatigue and muscle soreness scores in post-nap compared to both post-rest and pre-nap scores. The detailed results of Hooper’s subcategories, analyzed across different days, are presented in Table 2.

**TABLE 2:** Values (mean ± SD in arbitrary units) of Hooper’s sleep quality, stress, fatigue and muscle soreness scores across five days in NAP and CON.

		NAP		CON	

		Pre	Post	Pre	Post
Sleep quality	Day 1	2.92 ± 1.68	2.92 ± 2.02	3.00 ± 1.76	3.75 ± 1.54
	Day 2	3.67 ± 1.15	3.00 ± 0.85 * b	3.08 ± 1.78	4.33 ± 1.15
	Day 3	3.75 ± 1.22	2.58 ± 1.31 ** a, $ a	3.17 ± 0.72	4.83 ± 1.34 $$ a
	Day 4	3.25 ± 0.62	2.75 ± 0.75 * a	3.83 ± 1.90	4.42 ± 2.11 $ a
	Day 5	3.08 ± 1.38	3.00 ± 1.60 * a	3.33 ± 1.50	4.42 ± 1.38 $$ b

Stress	Day 1	1.75 ± 0.62	1.67 ± 0.65	2.08 ± 0.79	2.25 ± 0.62
	Day 2	2.42 ± 0.79	2.42 ± 0.79	2.50 ± 0.52	2.33 ± 0.49
	Day 3	2.75 ± 1.06	2.75 ± 0.62	2.67 ± 0.65	2.33 ± 0.78
	Day 4	3.00 ± 1.41	3.17 ± 1.27	3.58 ± 0.79	3.25 ± 0.75
	Day 5	3.25 ± 1.36	2.75 ± 0.75	2.75 ± 0.87	3.00 ± 1.41

Fatigue	Day 1	2.08 ± 0.51	1.92 ± 0.51	2.67 ± 0.78	2.83 ± 1.40
	Day 2	2.83 ± 1.34	2.25 ± 0.62 * a	2.75 ± 1.54	3.75 ± 1.54 $ a
	Day 3	3.83 ± 1.59	2.75 ± 1.54 $ a	3.50 ± 1.45	3.33 ± 1.15
	Day 4	3.83 ± 1.47	2.58 ± 1.24 * b, $ a	3.75 ± 1.54	4.17 ± 1.90
	Day 5	3.17 ± 1.34	2.92 ± 1.24 * b	3.25 ± 1.22	4.08 ± 1.51

Muscle soreness	Day 1	2.58 ± 1.88	2.00 ± 1.04 ** a	3.83 ± 1.80	4.17 ± 1.19
	Day 2	2.33 ± 1.61	2.58 ± 1.08 ** a	4.58 ± 1.16	4.42 ± 1.16
	Day 3	2.83 ± 1.34	3.00 ± 1.48	2.50 ± 0.80	2.33 ± 1.07
	Day 4	3.50 ± 1.68	2.67 ± 0.98 * a	4.25 ± 1.82	4.67 ± 1.78
	Day 5	4.17 ± 1.99	2.50 ± 1.45 ** a, $$ a	4.58 ± 1.68	4.75 ± 1.42

Abbreviations: CON: Five consecutive days of rest (control condition). NAP: Five consecutive days of napping. Pre: Before nap/rest period. Post: After nap/rest period. Significant difference compared to CON,

* p < 0.05,

** p < 0.01. Significant difference compared to pre- nap/rest,

$ p < 0.05,

$$ p < 0.01.

a and

b indicate large and medium Cohen’s *d* effect size, respectively.

#### Perceived exertion (RPE)

A significant effect of day was observed in RPE (F_(1,11)_ = 14.26, p = 0.003, ɳp2 = 0.56) during the RJT. However, no significant effects of condition (F_(1,11)_ = 0.21, p = 0.653, and ɳp2 = 0.02) and interaction (day × condition) (F_(1,11)_ = 1.89, p = 0.197, and ɳp2 = 0.15) were observed. Bonferroni post hoc test revealed that RPE scores decreased significantly on Day 5 compared to Day 1 in NAP (p = 0.002) (Table 3).

**TABLE 3:** Values (Mean ± SD) of jump height and relative power, and rating of perceived exertion (RPE) scores during the repeated jump test.

	Day 1		Day 5	

	NAP	CON	NAP	CON
Jump height (cm)	34.94 ± 7.02	35.01 ± 6.66	38.41 ± 6.86 ** a, ## a	35.25 ± 6.96
Power (W/kg)	36.97 ± 9.69	38.18 ± 8.25	41.66 ± 9.29 *** a, ## a	36.90 ± 8.89
RPE (a.u.)	14.17 ± 1.27	13.92 ± 1.83	11.83 ± 1.59 ## a	12.58 ± 1.78

Abbreviations: CON: Five consecutive days of rest (control condition). NAP: Five consecutive days of napping. RPE: Rating of perceived exertion. Significant difference compared to CON,

*p < 0.05,

** p < 0.01,

*** p < 0.001. Significant difference compared to Day 1 NAP,

#p < 0.05,

## p < 0.01.

a indicates large Cohen’s *d* effect size.

### Heart rate variability (HRV)

**FIG. 4:**
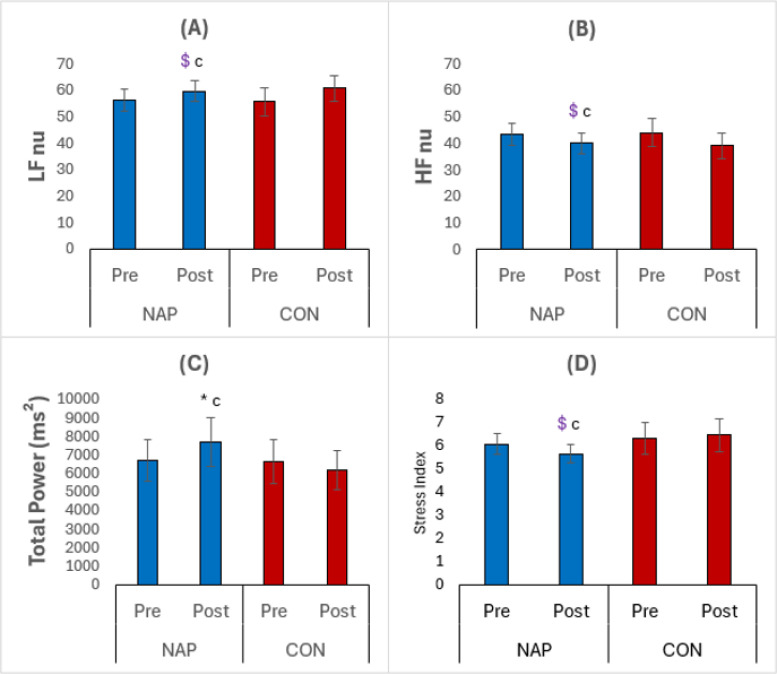
Five-day averaged mean ± standard error of heart rate variability parameters: (A) normalized unit (nu) of Low frequency (LF), (B) Hight frequency (HF), (C) Total power and (D) Stress index. CON: Five consecutive days of rest (control condition). HF: High frequency. LF: Low frequency. ms^2^: Milliseconds squared. NAP: Five consecutive days of napping. nu: Normalized unit. Pre: Before nap/rest period. Post: After nap/rest period. Significant difference compared to CON, *p < 0.05. Significant difference between pre- and post- nap/rest, $p < 0.05. c indicates small Cohen’s *d* effect size.

No significant changes were observed between conditions (NAP vs. CON) across the five days for HRmean, HRpeak, HRmin, SDNN, RMSSD, pNN50, or HRV index. For frequency domain parameters, the three-way repeated-measures ANOVA revealed only a significant main effect of time for the LFnu (F_(1,11)_ = 13.52, p = 0.004, ɳp2 = 0.55), HFnu (F_(1,11)_ = 13.66, p = 0.004, ɳp2 = 0.55) and LF/HF ratio (F_(1,11)_ = 10.12, p = 0.009, ɳp2 = 0.48). Post hoc analyses showed that the 5-day averaged values of LFnu and the LF/HF ratio increased (p = 0.026 and p = 0.015, respectively), whereas HFnu decreased (p = 0.026) from pre- to post-nap, while corresponding pre-to-post changes in the control condition did not reach statistical significance (all p ≥ 0.055) (Figure 4). Moreover, repeated measures ANOVA revealed a significant interaction (condition × time) for TP and stress index (F_(1,11)_ = 6.10, p = 0.031, and ɳp2 = 0.36 and F_(1,11)_ = 4.88, p = 0.025, ɳp2 = 0.38, respectively). Statistical analyses showed that the 5-day averaged TP values were significantly higher after the nap compared to after rest (p = 0.033) and that the stress index decreased significantly in post-nap compared to pre-nap (p = 0.030) (Figure 4). More particularly on Day 5, the stress index was significantly lower post-nap compared with pre-nap (5.60 ± 1.45 *vs*. 6.82 ± 1.59, respectively; p = 0.017, *d* = 0.81).

### Basketball-specific physical performance

#### Offensive agility (OA)

**FIG. 5:**
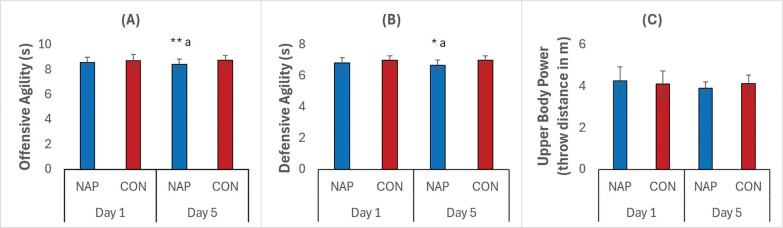
Mean values (± SD) of (A) offensive agility, (B) defensive agility and (C) upper body power in NAP and CON conditions on Day 1 and Day 5. CON: Five consecutive days of rest (control condition). m: Meters. NAP: Five consecutive days of napping. s: Seconds. Significant difference compared to CON, *p < 0.05, **p < 0.01. a indicates large Cohen’s *d* effect size.

ANOVA tests revealed a significant effect of condition (F_(1,11)_ = 9.41, p = 0.011, ɳp2 = 0.46) but a non-significant effect of day (F_(1,11)_ = 0.84, p = 0.379, ɳp2 = 0.07) and interaction (day × condition) (F_(1,11)_ = 1.03, p = 0.331, ɳp2 = 0.09) for offensive agility. The Bonferroni post hoc tests revealed no significant difference on Day 1 (p = 0.328). However, NAP showed a significantly better performance compared to CON on Day 5 (p = 0.005) (Figure 5).

#### Defensive agility (DA)

ANOVA showed a significant effect of condition (F_(1,11)_ = 5.29, p = 0.042, ɳp2 = 0.33), but no significant effect of day (F_(1,11)_ = 2.46, p = 0.145, ɳp2 = 0.18) or interaction was found (F_(1,11)_ = 4.81, p = 0.051, ɳp2 = 0.30) for the defensive agility. Post hoc tests showed a significant improvement (p = 0.015) in NAP on Day 5 compared to CON on the same day (Figure 5).

#### Upper body power (UBP)

ANOVA showed no significant main effects of condition (F_(1,11)_ = 2.99, p = 0.112, ηp^2^ = 0.21) or day (F_(1,11)_ = 0.10, p = 0.754, ηp^2^ = 0.01), nor a significant interaction (F_(1,11)_ = 4.51, p = 0.057, ηp^2^ = 0.29) for UBP (Figure 5).

#### Repeated jumping performance (RJ)

Two-way repeated-measures ANOVA revealed a significant effects of condition (F_(1,11)_ = 5.22, p = 0.043, ɳp2 = 0.32) and a significant condition × day interaction (F_(1,11)_ = 12.15, p = 0.005, ɳp2 = 0.53) for jump height. For relative power during the RJT, a significant interaction (F_(1,11)_ = 27.57, p = 0.0002, ɳp2 = 0.72) was found. The Bonferroni post hoc analyses showed significantly better performance on Day 5 in NAP compared to CON for both jump height (p = 0.005) and relative power (p = 0.0001). Moreover, significant increases from Day 1 to Day 5 were observed in NAP for jump height (p = 0.007) and relative power (p = 0.008). No significant changes were found between Day 1 and Day 5 in CON for jump performance (i.e., jump height and power). (Table 3).

## DISCUSSION

This study is the first to assess the effectiveness of repeated daytime napping during a five-day training camp in adolescent basketball players. The main findings showed that NAP significantly increased 24-h TST compared to CON. Additionally, it enhanced basketballspecific physical performance (i.e., offensive/defensive agility, repeated jumps) and improved perceptual measures (i.e., sleep quality, fatigue, muscle soreness, RPE) and physiological responses.

A major result in the present study is that, although nocturnal sleep duration did not differ significantly between conditions over the five days, napping helped athletes meet the recommended sleep duration for adolescents (8–10 hours) [43], with a 5-day averaged TST over 24 h of 8.4 hours in NAP, while CON remained below recommendations at 7.7 hours. These findings align with those of a previous study, which reported that despite a reduction of 81 min in nocturnal sleep during a two-week training camp, athletes who napped increased their total sleep by 30 min [20]. Given that adolescent athletes often experience compromised sleep during training camps [10, 44], and considering the detrimental effects of insufficient sleep on performance and injury risk [8], our findings may help to prevent the deleterious effects of inadequate sleep on athletes’ health and steer clear of injuries. Furthermore, as previously reported [45], when implemented in a team-sport environment, naps should balance the need to enhance performance while not disturbing subsequent sleep patterns, as this could hinder the recovery process after training or competition [45]. Interestingly, the 60-min daytime nap opportunity did not negatively impact nocturnal sleep quality or duration throughout the 5-day micro-cycle, with SOL even being significantly lower on Day 2 in the NAP condition compared to CON.

Furthermore, the findings of this study suggest that a 60-min nap opportunity implemented across a five-day micro-cycle was associated with selective changes in HRV parameters. Specifically, total power increased following the nap condition compared with the control rest condition, accompanied by a pre- to post-nap reduction in the stress index, particularly on Day 5. These changes suggest an overall enhancement of cardiac autonomic variability [46, 47] and a reduction in physiological stress following repeated napping, supporting a potential cumulative recovery-related effect. These stress index results are in line with previous work investigating the acute napping effect in high-level adult athletes [48]. Moreover, a pre-topost decrease in the 5-day averaged HFnu values, alongside increases in the 5-day averaged LFnu values and the LF/HF ratio, was observed, reaching significance in the NAP condition but not in CON. This pattern could be indicative of a transient post-nap sympathetic reactivation, potentially preparing the body for a much-required sympathetic comeback following peaceful rest [47, 48]. However, interpretation of these indices requires caution. As highlighted by Billman et al. [49], the LF/HF ratio may not reliably reflect cardiac sympatho-vagal balance due to its sensitivity to respiratory influences, prevailing heart rate, baroreflex modulation and mathematical coupling inherent to normalized units, as well as the fact that LF power does not represent a purely sympathetic component [49]. Accordingly, frequency-domain HRV indices in the present study are discussed descriptively rather than as direct indicators of autonomic balance. Importantly, although parasympathetic predominance during daytime sleep, and particularly during NREM sleep, is well established and has been previously described as a state of cardiovascular “quiescence” or a “cardiovascular holiday” [46, 48, 50], HRV was not recorded during the nap itself in the present study, but rather before and immediately after the nap opportunity. Consequently, sleep-stage–specific autonomic responses cannot be directly used to explain post-nap HRV findings. Any transient post-nap autonomic shifts may instead reflect short-term arousal associated with the sleep-wake transition rather than recovery itself. The absence of significant changes in several time-domain HRV parameters (e.g., higher SDNN in NAP *vs*. CON) further supports a cautious interpretation of the frequency-domain findings and highlights the need for studies with larger sample sizes and more comprehensive autonomic assessments. Taken together, while repeated daytime napping appears to beneficially influence global HRV and stress-related indices over a short-term micro-cycle, mechanistic interpretations based on LF, HF, or LF/HF should remain conservative, and future research should further explore the temporal dynamics and functional relevance of post-nap autonomic responses.

Moreover, one of the key findings of this study is that five consecutive days of nap opportunities led to significant improvements in basketball-specific physical performance, with increases in offensive and defensive agility, as well as jump relative power and height compared to CON. Recent research has explored the impact of napping on sportspecific performance [17, 51], demonstrating improved agility in volleyball [51] and basketball players [17]. Unlike the present study, these investigations focused only on acute effects rather than repeated naps and were conducted exclusively with adult athletes. A plausible explanation for the improvement in physical performance observed with napping in the present study could be the increased total sleep duration over 24 h, which may enhance recovery and better prepare athletes for high-intensity efforts, as sleep, particularly nonrapid eye movement (NREM) sleep, plays a crucial role in physical restoration and repair [52]. In this context, a recent systematic review suggested that the benefits of napping may be linked to the metabolic recovery associated with longer nap durations, having a more significant slow-wave component of sleep [53]. Therefore, the findings of the present study are meaningful for optimizing basketball-specific performance in adolescents, as they demonstrate higher agility and repeated jump ability, which could ultimately contribute to greater competitive success.

However, contrary to our expectations, basketball-specific physical performances in the adolescent athletes of the present study did not improve following a single nap (Day 1) compared to CON. These findings align with those of Suppiah et al. [22], who reported that a single 30-min nap did not significantly enhance 2 m, 10 m, or 20 m average sprint times in adolescent athletes. In the present study, napping-related improvements in basketball-specific performances were only observed on Day 5. In the same way, in NAP condition, jump height and power were significantly higher on Day 5 compared to Day 1, and were accompanied by a 16.5% reduction in RPE. Therefore, while athletes may not have required napping on Day 1, the progressive accumulation of training fatigue [9, 10] likely increased the physiological need for daytime sleep suggesting that repeated naps played a key role in counteracting accumulated fatigue and optimizing recovery during prolonged training periods. Moreover, the discrepancy between the effects of a single nap and consecutive naps may be explained by a progressive adaptation to napping, leading to improvements in nap quality and duration throughout the micro-cycle. Given that the participants of the present study were not habitual nappers, the first nap may have been of insufficient quality (high SFI≈53%) and duration (TST≈33 min) to induce measurable gains in sport-specific abilities. In this regard, future studies may benefit from including an actual nap during the familiarization session, rather than limiting familiarization to exposure to the napping environment. Notably, participants progressively optimized their daytime sleep over the five-day training camp, as evidenced by significant increases in naps’ TST on Days 3 and 4 compared to Day 1, along with significant reductions in SFI on Days 4 and 5 compared to Day 2. These results suggest an adaptation to napping, allowing athletes to maximize the beneficial effects of daytime sleep reported previously [14, 15]. In this context, it has been shown that elite athletes have a superior ability to initiate sleep during a daytime nap compared to non-athletes [54].

Furthermore, the improved physical performance observed with napping in this study was accompanied by reduced subjective fatigue (Days 2, 4, and 5) and muscle soreness (Days 1, 2, 4, and 5) compared to CON, suggesting that napping alleviates training-induced discomfort. Given that athletes in training camps often experience pain and soreness, which can impair recovery and performance [11, 12], these findings further support napping-induced hypoalgesic effect [17, 24]. This is particularly relevant for jump performance, as repeated jumping is a fatiguing task [55] and may be more sensitive to perceived effort, fatigue, and muscle soreness. Additionally, the progressive improvement in Hooper’s global score throughout the micro-cycle highlights the cumulative benefits of repeated napping, with consistently lower post-nap scores compared to post-rest, indicating better recovery and readiness and highlighting repeated napping’s potential as a recovery strategy.

### Strengths and limitations

To the best of the authors’ knowledge, this study is among the first to examine the effects of repeated daytime napping across a five-day micro-cycle in adolescent basketball players. Conducted during a real training camp, it used basketball-specific performance tests and combined objective sleep monitoring (actigraphy), subjective recovery measures, physiological markers, and physical performance outcomes, thereby enhancing ecological validity and practical relevance.

However, the present study has some limitations. First, sleep assessment using actigraphy does not provide information on sleep architecture; therefore, the presence and amount of slow-wave sleep during the nap opportunities could not be verified. Second, the intervention period was limited to five consecutive days, which constrains inference regarding longer-term effects. Third, in the NAP condition, HRV was assessed only before and after the nap opportunity, with no measurements obtained during sleep. Consequently, the timing of post-nap recordings relative to awakening may have influenced frequency-domain indices. Moreover, LF, HF, and the LF/HF ratio, particularly when expressed in normalized units, should be interpreted with caution and along with time-domain HRV indices. Finally, the relatively small sample size and the inclusion of male athletes only may limit the generalizability of the findings. Although a posteriori statistical power was high (≥ 0.98) for most primary outcome variables (i.e., defensive agility, upper body power, jump height and relative power), power was lower for offensive agility (0.48), and these results should therefore be interpreted with caution. Future studies should include larger, mixed-sex samples, extend the intervention period as well as the washout duration to better control for potential carryover or withdrawal effects, and use EEG-based devices to monitor sleep architecture during both daytime naps and nocturnal sleep.

### Practical applications

Implementing repeated daytime nap opportunities across consecutive days may represent a practical, non-invasive recovery strategy for adolescent athletes during congested training periods. In non-habitual nappers, benefits are more likely to emerge after several consecutive days of napping rather than following a single nap. Specifically, scheduling a 60-min nap opportunity early in the afternoon across consecutive days may help increase total 24-h sleep duration and support perceived recovery in applied settings such as training camps or intensive micro-cycles. In this study, repeated napping was associated with improvements in basketball-specific physical performance outcomes and with favorable changes in perceptual parameters and selected physiological indices (e.g., total HRV power and stress index). Coaches, scientists and practitioners should consider these findings as preliminary and integrate structured nap opportunities as part of a broader recovery plan, particularly for athletes with habitual sleep restrictions.

## CONCLUSIONS

The present findings suggest that the potential benefits of daytime nap opportunities in adolescent athletes are primarily observed when naps are implemented repeatedly across consecutive days during an intensive training micro-cycle, rather than following a single nap session. In this context, a five-day schedule of 60-min early-afternoon nap opportunities was associated with improvements in selected sport-specific performance measures and favourable changes in global HRV/stress-related indices. However, given the short duration of the intervention and the limitations inherent to frequency-domain HRV interpretation, these findings should be considered preliminary. Moreover, further studies are needed to clarify the mechanisms underlying the benefits associated with repeated daytime napping.
